# Devices for dosimetric measurements and quality assurance of the Xstrahl 300 orthovoltage unit

**DOI:** 10.1002/acm2.13220

**Published:** 2021-03-18

**Authors:** Tze Yee Lim, Dragan Mirkovic, Xin Wang, Ramesh Tailor

**Affiliations:** ^1^ Department of Radiation Physics The University of Texas MD Anderson Cancer Center Houston TX USA

**Keywords:** kilovoltage x‐ray, orthovoltage, QA

## Abstract

The Xstrahl 300 orthovoltage unit is designed to deliver kilovoltage radiation therapy using the appositional technique. However, it is not equipped with some typical linear accelerator features, such as mechanical distance indicator and crosshair projection, which are useful for facilitating equipment setup during various quality assurance (QA) and research activities. Therefore, we designed and constructed slip‐in devices to facilitate QA for dosimetric measurements of our Xstrahl 300 unit. These include: (a) an ion chamber positioning system for dosimetric measurements, (b) a mechanical pointer for setting dosimeter distance to a nominal 50 cm, and (c) a crosshair projector with built‐in light to facilitate alignment of dosimeter to the center of the radiation field. These devices provide a high degree of setup reproducibility thereby minimizing setup errors. We used these devices to perform QA of the Xstrahl 300 orthovoltage unit. One of the QA tests we perform is a constancy check of beam output and energy. Our data since start of clinical use of this unit (approximately 2.5 yr) show dose outputs to be remarkably reproducible (2σ = ±0.4%) for all three clinical beams (75, 125, and 250 kVp). These devices have provided both convenience and high‐precision during the unit’s commissioning, and continue to provide the same for various QA activities on the Xstrahl 300 orthovoltage unit.

## 1 INTRODUCTION

Xstrahl 300 (Xstrahl Ltd., Surrey, UK) is a commercial orthovoltage unit used for radiation therapy. It is capable of delivering kilovoltage x‐ray beams of 40–300 kVp.^1^ Radiation beams with kilovoltage x‐ray energies deposit most of the dose close to the skin.^2^, ^3^ Consequently, kilovoltage x‐ray beams are suited for treatment of shallow lesions, such as nonmelanoma skin cancer.^3^, ^4^, ^5^ For treatment using the appositional technique, the ends of fixed‐length applicators are placed against the patient’s skin. The applicators can have square or round apertures of varying sizes, and their ends may be open or closed.^6^ The clinically desired depth of skin penetration dictates the choice of beam energy.

To ensure safe and consistent radiation therapy, routine quality assurance (QA) tests are essential. The Xstrahl 300 orthovoltage unit does not come equipped with mechanical distance indicators, crosshair projection light field, or wall lasers. These are common devices or features of linear accelerators in radiation oncology clinics that are used for equipment positioning during QA. Precision in dosimeter positioning during any dosimetry measurement is an important factor in reducing measurement uncertainty, thereby enhancing identification of machine‐related problems or dosimetry issues. Kilovoltage x‐ray beam dosimetry is also highly sensitive to measurement distances,^7^ further emphasizing the importance of precise dosimeter positioning.

To achieve precise dosimeter positioning during QA measurements of our Xstrahl 300 orthovoltage unit (output, energy, cone factor, etc.), an ion chamber positioning system was designed and constructed. A couple of other slip‐on devices, namely a mechanical distance pointer and crosshair projector, were also designed and constructed to facilitate QA measurements in general. Herein, these QA devices used with our Xstrahl 300 orthovoltage unit are described, and the results of QA tests performed with them are reported. The devices can aid radiation oncology clinics in complying with national and international standards for QA of kilovoltage x‐ray radiotherapy equipment. While the American Association of Physicists in Medicine (AAPM) has not published QA recommendations for kilovoltage x‐ray machines, the report by Task Group 61 (TG‐61) states that “the consistency of the x‐ray output shall be checked routinely”.^8^ The ion chamber positioning system can be used to perform routine checks of the x‐ray output. Output checks are also recommended in the comprehensive technical quality control guidelines for kilovoltage x‐ray radiotherapy machines published by the Canadian Partnership for quality radiotherapy (CPQR).^9^

In addition to the typical radiation oncology clinic setting, the devices described here can be adapted to facilitate other types of QA or research in various settings, such as outpatient dermatology clinics or settings with limited resources. A recently published consensus dermatology guidelines described the renaissance of kilovoltage x‐ray radiotherapy for the treatment of nonmelanoma skin cancers and recurrent keloids.^5^ This may be because kilovoltage x‐ray radiotherapy has produced better cosmesis for certain anatomical locations than has surgery, is more cost‐effective than electronic brachytherapy,^10^ and has a sharper lateral penumbra than does electron beam therapy.^5^ Furthermore, in low‐income countries, the frequent trade‐off in determining the appropriate level of radiotherapy is that between evidence of clinical efficacy and treatment complexity, the latter of which gives rise to personnel training issues, high machine cost, maintenance challenges, etc.^11^ Thus, kilovoltage x‐ray treatment, which does not require complex treatment plans, is especially relevant for low‐income countries, as it is one of the few radiotherapy techniques for which clinical efficacy^4^, ^5^ aligns with cost‐effectiveness and ease of use.^1^, ^10^ Moreover, the use of kilovoltage x‐ray beams for radiobiology research has been rising.^3^, ^12^, ^13^, ^14^ 25.0 % of radiobiology studies used kilovoltage x‐rays as the radiation source, according to a large‐scale systematic review of radiobiology preclinical and translational research literature over the last two decades.^13^ In recent years, kilovoltage x‐rays have even outpaced Co‐60 and Cs‐137 gamma rays to become the most prevalent radiation type used in murine research.^14^

To facilitate QA or research in different settings, structural or procedural modifications to the use of the devices presented in this paper can be made. For instance, the ion chamber positioning device was designed to hold a Farmer chamber, but can be used with any cylindrical ion chamber with a diameter <1.5 cm, and furthermore, the user may even fashion and attach different holders, such as a platform to hold small animals or cells for preclinical research. On the other hand, the mechanical distance pointer can be used whenever positioning at 50 cm distance is desired, such as during transmission measurements of an eye shield to navigate around the shield’s suture hole knob, while the crosshair projector can be used to check centering of objects in most situations. This report may inspire others to adapt the designs described here and construct similar devices to suit their specific QA or research activities on their kilovoltage x‐ray units.

## 2 MATERIALS AND METHODS

### 2.A Construction of the QA devices

**Fig. 1:**
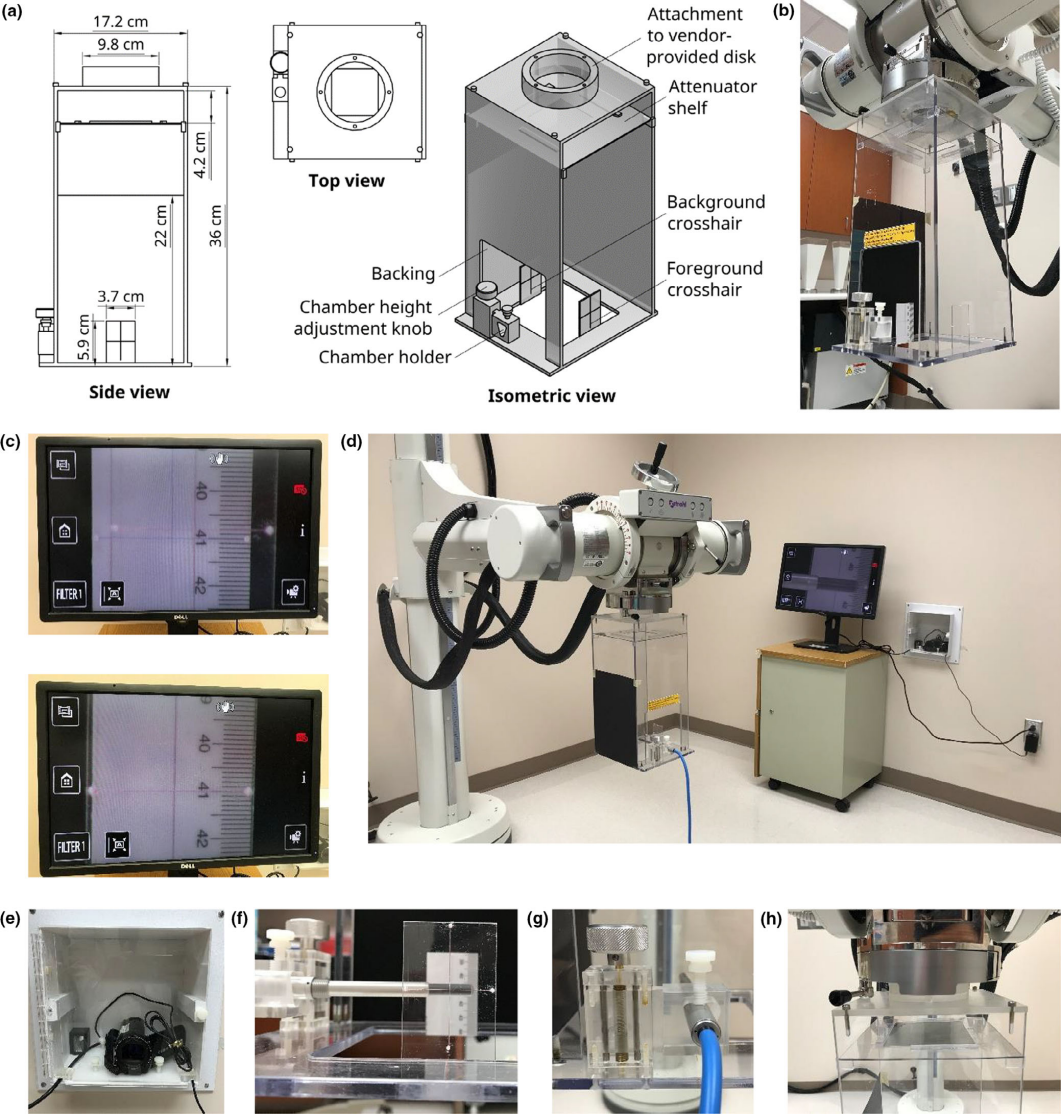
The ion chamber positioning system we created for the Xstrahl 300 orthovoltage unit. (a) Dimensions, different views, and various parts of the ion chamber positioning device. (b) The ion chamber positioning device mounted in the Xstrahl 300 orthovoltage unit. (c) The ion chamber positioning device was adjusted such that the blue and red crosshairs moved from not aligned (top) to aligned (bottom) as viewed through the camera. (d) The ion chamber positioning device and camera setup for the ion chamber positioning system. (e) The camera was encased and mounted on a wall to allow freedom of movement around the Xstrahl 300 system. (f) A Farmer chamber placed in the ion chamber positioning device. (g) The Farmer chamber was secured within the chamber holder, and the distance from the midline of the chamber to the horizontal lines of the crosshairs was minimized using the height adjustment knob. (h) Different attenuators would be placed in the attenuator shelf for transmission measurements for different energies.

As shown in Fig. 1, the ion chamber positioning system consisted of an ion chamber positioning device and a camera setup. The frame of the ion chamber positioning device was constructed from polycarbonate sheets attached to each other using screws and solvent‐bonded using dichloroethylene. Near the bottom of the ion chamber positioning device is a channel that allows a cylindrical ion chamber to pass through. To ensure consistent focus to skin distance (FSD), a small height adjustment knob next to the channel can be used to vertically adjust the chamber 0.91 mm per full knob turn. Near the top of the ion chamber positioning device is a shelf that allows for placement of beam attenuators upstream of the chamber. A field‐defining disk can be slipped onto the top of the ion chamber positioning device. For QA measurements in our clinic, a field‐defining disk obtained from the orthovoltage unit manufacturer that projected a 10 cm × 10 cm square field was used.

To achieve the desired positioning accuracy, a magnified view of the chamber position and reference lines was required. To magnify the view of the chamber, a camera with magnification capability was encased in a box with a transparent front and permanently mounted on a wall. The camera was mounted approximately 1.8 m away from the typical location of the ion chamber positioning device to minimize disturbance to the camera as well as the clinical workflow. The camera was connected to a power outlet and a monitor that displayed the camera view. The 10 × magnification view showed the ion chamber and two sets of crosshairs. These two sets of crosshairs served as reference lines to ensure consistent chamber positioning month to month. The first set of crosshairs was defined in the background of the ion chamber and consisted of two thin blue plastic threads affixed perpendicular to each other on a plastic ruler. The second set of crosshairs was defined in the foreground of the ion chamber and consisted of two thin red plastic threads affixed perpendicular to each other on a small polycarbonate sheet. Matching up both crosshair sets’ vertical lines ensured that the dosimeter was aligned along the beam central axis, and matching up their horizontal lines ensured consistent positioning [Fig. 1(c)]. The positioning of the chamber along the camera’s line‐of‐sight is fixed since the chamber holder is affixed on the ion chamber positioning device [Fig. 1(d)]. Furthermore, the vertical line in the foreground of the chamber can be used to guide horizontal centering during insertion of the chamber [Fig. 1(f)], while the horizontal line in the foreground of the chamber can be used to check the chamber’s pitch and guide vertical centering of the chamber using the height adjustment knob [Fig. 1(g)]. A thin black piece of cardboard was taped behind the background crosshair to ease camera focusing.

**Fig. 2:**
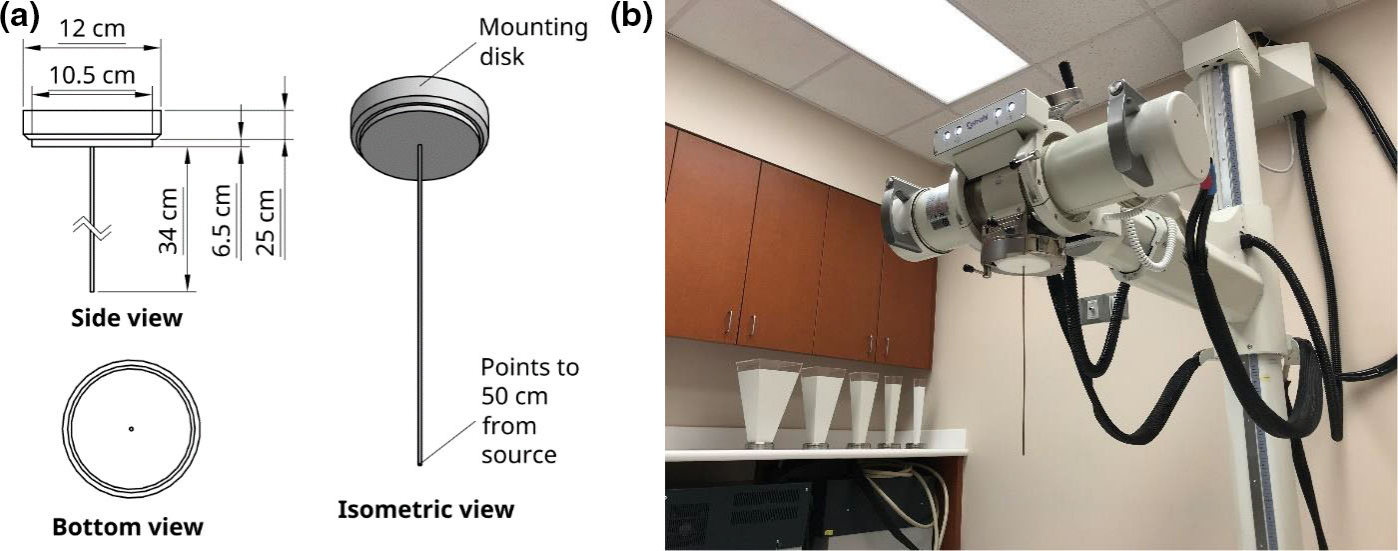
The mechanical distance pointer we created for the Xstrahl 300 orthovoltage unit. (a) Dimensions, different views, and various parts of the mechanical distance pointer. (b) The mechanical distance pointer mounted in the Xstrahl 300 orthovoltage unit. The tip of the pointer indicated 50 cm from the focal spot of the x‐ray tube. Bulky standard applicators of different field sizes, also of 50 cm focus to skin distance, can be seen on the back counter.

**Fig. 3:**
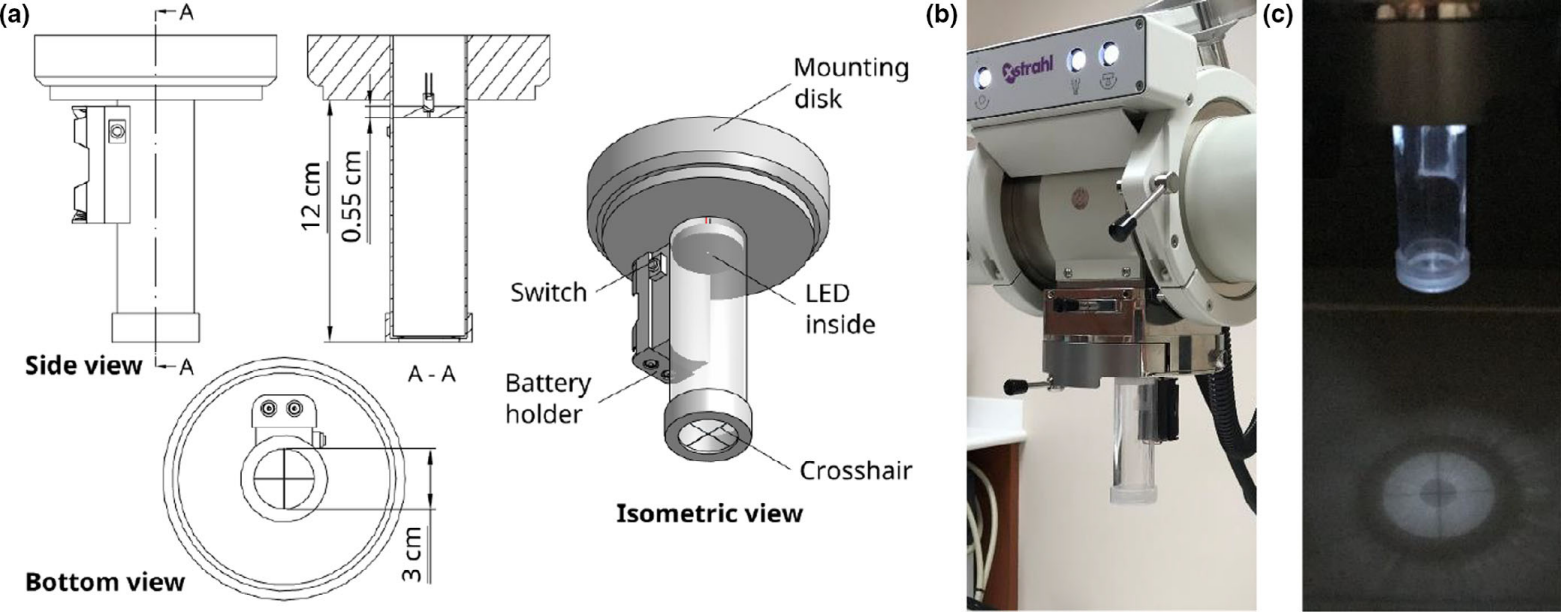
The crosshair projector we created for the Xstrahl 300 orthovoltage unit. (a) Dimensions, different views, and various parts of the crosshair projector. (b) The crosshair projector mounted in the Xstrahl 300 orthovoltage unit. (c) Projection of the crosshair shadows with the light‐emitting diode turned on.

Other supporting devices that we devised to facilitate QA of the Xstrahl 300 orthovoltage unit were a mechanical distance pointer and crosshair projector. The mechanical distance pointer consists of a brass rod in a plastic disk that can be mounted onto the unit as shown in Fig. 2. To assemble the pointer, the rod was force‐fitted into the tight hole at the center of the disk. In use, the tip of the rod indicated 50 cm FSD when mounted in the Xstrahl 300 orthovoltage unit’s sub‐tube assembly. The crosshair projector consisted of two thin metal wires attached perpendicular to each other on the base of a transparent plastic cylinder, an attached light‐emitting diode, a battery for the light‐emitting diode, and a plastic disk that can be mounted onto the orthovoltage unit (Fig. 3). This device effectively mimicked the built‐in light field with crosshair projection on a linear accelerator. It is useful in aiding the positioning of phantoms and dosimeters, such as ion chambers, film, thermoluminescent dosimeters, and optically stimulated luminescent dosimeters.

### 2.B Measurements performed using the QA devices

One of the QA tests performed for the Xstrahl 300 orthovoltage unit was measurement of the various beam energies' output. First, the ion chamber positioning device was mounted onto the orthovoltage unit. To measure the radiation dose output, a Type 30013 Farmer Chamber (PTW, Freiburg, Germany) was used.^8^, ^15^ The ion chamber was inserted into the channel of the chamber holder and connected to an electrometer (Model 602; Keithly Instruments, Inc., Cleveland, OH). The camera and connected monitor were then switched on. The orthovoltage unit tube assembly with the mounted ion chamber positioning device was moved into position so that the chamber could be seen on the camera view. Next, both sets of crosshairs were aligned by rotating the ion chamber positioning device and adjusting the tube assembly height as necessary. The chamber positioning was adjusted using the height adjustment knob. The room temperature was measured using a thermometer, and the room’s atmospheric pressure was measured using a barometer. At our institution, three orthovoltage energies were commissioned: 75, 125, and 250 kVp. These energies were chosen based on clinical experience and physician preference. Two output readings were obtained for each energy. The measured outputs, corrected for temperature and pressure variations, were compared with baseline outputs to determine the output deviations. Finally, the monthly output deviations over 2.5 yr were plotted. This output check was performed more than once a month in the initial months following commissioning of the Xstrahl 300 orthovoltage unit, yielding 35 data points in total.

Another QA test performed was the energy consistency check. Designated filters were placed in the filter slot of the orthovoltage unit, and additional attenuators were placed on the shelf of the ion chamber positioning device [Fig. 1(h)]. Three metal sheets—1.8 mm thick aluminum, 2.8 mm aluminum, and 1.27 mm copper—were used for transmission measurements of the 75, 125, and 250 kVp beams respectively. The thicknesses of the additional attenuators were chosen to sample a region within the 40–60% transmission zone as an indicator of the energy spectrum’s stability. To measure the transmitted radiation dose output, the Farmer chamber was placed in the ion chamber positioning device downstream from the attenuators. The monthly measured transmissions were compared with the baseline transmissions and plotted over 2.5 yr.

In addition, we obtained an independent output check of the Xstrahl 300 orthovoltage unit from The MD Anderson Radiation Dosimetry Services (RDS).^16^ After placing the thermoluminescent detectors (TLDs) on the folding platform provided by RDS, the crosshair projector was mounted such that the TLDs could be centered. Next, the mechanical distance pointer was mounted to guide positioning of the TLDs at 50 cm FSD. The slender profile of the mechanical distance pointer allowed for it to then be removed without disturbing the TLDs. After dose delivery with 250 kVp, the TLDs were sent back to RDS. RDS later provided us with a report of the independent dose measurement.

## 3 RESULTS

We used the ion chamber positioning system, mechanical distance pointer, and crosshair projector to achieve precise positioning of the chamber during QA tests of our Xstrahl 300 orthovoltage unit.

**Fig. 4:**
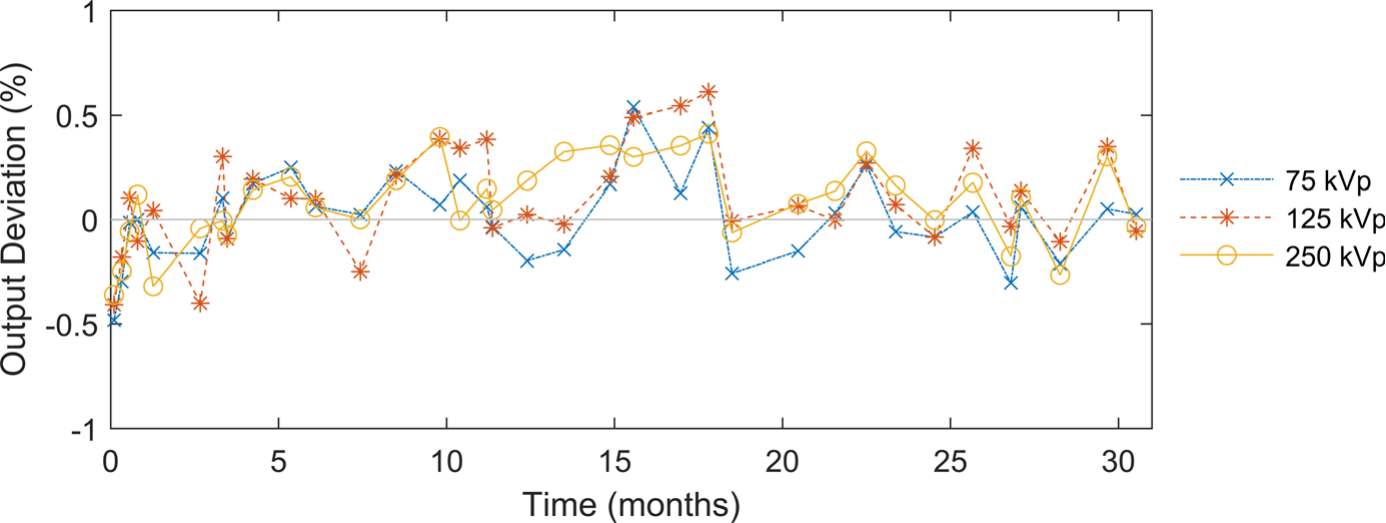
Radiation output measurements using the ion chamber positioning system with the Xstrahl 300 orthovoltage unit over 2.5 yr.

The radiation output deviations from baseline for the three energies on our Xstrahl 300 orthovoltage unit over 2.5 yr as measured using the ion chamber positioning system are shown in Fig. 4. The mean (±standard deviation) output reproducibility across all energies was 0.06% ± 0.22% (range, −0.48% to 0.61%). We observed no radiation dose output drift. Use of the aforementioned QA devices greatly reduced setup uncertainty and enabled us to precisely monitor the radiation output.

**Fig. 5:**
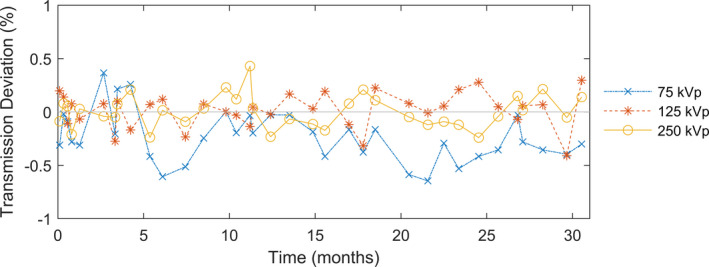
Radiation transmission measurements using the ion chamber positioning system with the Xstrahl 300 orthovoltage unit over 2.5 yr.

The transmission deviations from baseline for the three energies on our Xstrahl 300 orthovoltage unit over 2.5 yr as measured using the ion chamber positioning system are shown in Fig. 5. The mean (±standard deviation) transmission deviation across all energies was −0.07% ± 0.22% (range, −0.65% to 0.43%).

For the independent output check using TLDs from RDS set up using the mechanical distance pointer and crosshair projector, we obtained a ratio of output measurement performed by RDS to that performed by our institution of 0.98 (−2%), which was within the ±10% considered to be a satisfactory output check for orthovoltage energies by RDS.^16^

## 4 DISCUSSION

Various devices to aid reproducible QA testing of the Xstrahl 300 orthovoltage unit was constructed. We chose the construction materials for these QA devices based on availability, ease of construction, resistance to impact, and cost. For the ion chamber positioning device, the main reason for choosing polycarbonate was practicality. Polycarbonate can better withstand repeated handling over time than can acrylic, which may crack if mishandled. In addition, the transparency of polycarbonate makes the ion chamber easily visible during setup. The material also must be transparent to the radiation beam to allow the radiation beam to pass through it as well to prevent additional scatter. We used a minimum amount of polycarbonate material to reduce dose interference while maintaining solidity. Similarly, for the crosshairs, instead of metallic wires, we used thin plastic threads to prevent additional scatter. We initially used human hairs for the crosshairs, but keeping those fine crosshairs in focus via the camera viewport was difficult. We could consistently obtain well‐focused images with the thin plastic threads. As for the mechanical distance pointer, we considered stainless steel but ultimately chose brass because it was easier to file to the desired length. Lastly, for the crosshair projector, we chose a light‐emitting diode with sufficient brightness but that can still serve as a point source to ensure that the shadows of the crosshairs have a sharp penumbra.

We found the x‐ray outputs (output deviation = 0.06% ± 0.22%) and energies (transmission deviation = −0.07% ± 0.22%) to be very consistent over 2.5 yr of use in a radiation oncology clinic setting. These results reflected both the stability of the orthovoltage unit and the precision of the QA setup. The similarity of the uncertainty in the output measurements to the uncertainty in the transmission measurements suggests that we approached the limit of setup uncertainty of our system. Together with the magnification system, the ion chamber positioning device we employed enabled positioning of dosimeters to an accuracy of 0.1 mm. The ion chamber positioning device was primarily designed for efficient checks of dose outputs and beam energy. The high precision provided was a bonus. Nevertheless, this precision of our QA setup minimizes measurement uncertainties and allows us to better isolate machine‐specific issues, such that we can set a limit tighter than a typical action level of 3% deviation^8^ to trigger investigation of the accuracy of the tube potential and filament settings. Moving forward, we will continually monitor the orthovoltage unit’s output with the aforementioned QA devices over many years to ensure accurate delivery of radiation therapy.

Other devices could have been used to perform the tests described herein. For instance, a vendor‐provided fixed‐length applicator can be used instead of the ion chamber positioning device for the output measurements. In that event, because the effective point of measurement is at the center of the ion chamber’s sensitive volume^8^ and intersecting the applicator end into the chamber is not physically possible, correction factors may need to be applied to account for the added distance of the effective point of measurement to the end of the applicator and end‐plate scattering contribution if using closed‐ended applicators.^17^ In addition, our ion chamber positioning device has a shelf that can accept any custom attenuator for tailored transmission measurements. Due to these advantages, we designed these devices specifically to facilitate our QA needs. Similarly, a 50 cm fixed‐length applicator can be used for distance indications instead of the mechanical distance pointer described herein. From our experience, however, the light weight and slim profile of the mechanical distance pointer make for much easier handling and maneuvering around measurement systems.

The design of the ion chamber positioning device has some limitations. The ion chamber positioning device does not allow for concurrent use of an applicator, hence, any measurements performed with it will not account for the presence of an applicator. For instance, the output constancy measurements described here do not account for any changes to the applicator over time. Most clinics will use multiple applicators and measurements in reference conditions performed with a reference applicator can only monitor changes to one reference applicator. In our clinic, we use three sets of applicators with our Xstrahl 300 unit: closed‐ended square applicators provided by Xstrahl Ltd., as well as shorter open‐ended circular flat applicators and beveled applicators that used to be part of a decommissioned RT250 unit (Philips, Amsterdam, Netherlands). The latter ones are still used due to long‐standing clinical experience of our physicians and a slide‐in adaptor is used to mount these applicators. Given the three different sets of applicators, the initial motivation for designing the ion chamber positioning device was to be able to efficiently perform QA of the beam independent of the choice of applicator set. Clinical dosimetry for any kilovoltage x‐ray beam primarily involves the in‐air dose output in reference geometry, cone factor, and backscatter factor, among other factors.^3^, ^6^, ^18^ The cone factors and backscatter factors are not expected to change as long as the applicators do not get damaged. To monitor the integrity of all our applicators over time, we measure the cone factor with respect to our open reference geometry for each of our applicators annually. The backscatter factors are established based on data from TG‐61.^8^ Thus, the remaining variable is the in‐air dose output in reference geometry that reflects the machine performance. With the assistance of the described devices, we check this monthly via the described output and energy constancy tests as well as annually following the TG‐61 protocol^8^ for dose output and HVL measurements for beam energy. Again, we stress that since the ion chamber positioning device does not incorporate an applicator, it should serve as a reminder to separately account for the different applicators in clinical calculations to avoid incidents such as the Ottawa Orthovoltage Incident^19^ where backscatter factors for field sizes other than 10 × 10 cm^2^ were not applied, resulting in under‐ and over‐doses to patients. Another limitation of the ion chamber positioning device is that the described energy constancy check is done in the broad beam geometry. For measurement of definitive HVL, narrow beam geometry should be employed to minimize detection of radiation scattered from the attenuators.^3^, ^8^ While the ion chamber positioning device is not used during our annual check of the HVL, the mechanical distance pointer and crosshair projector are helpful to ensure correct chamber positioning in the radiation field under the scatter‐free narrow beam geometry.

## 5 CONCLUSIONS

Devices that facilitate QA tests of the Xstrahl 300 orthovoltage unit were designed and constructed. The detailed descriptions of these devices may inspire other clinics with kilovoltage x‐ray machines to construct similar devices of their own to support their various QA or research objectives.

## CONFLICT OF INTEREST

No conflict of interest.
